# ‘Aches and Pains’ of Filipino Migrant Workers in Malaysia: A Profile of Work-Related Musculoskeletal Disorders

**DOI:** 10.29024/aogh.2331

**Published:** 2018-08-31

**Authors:** Hernan C. Labao, Erwin M. Faller, May Florence D. Bacayo

**Affiliations:** 1 Global Health Network, Shah Alam, Selangor, MY; 2 School of Pharmacy, Management and Science University, Shah Alam, Selangor, MY; 3 Lecturer, Physiotherapy Department, Faculty of Health and Life Sciences, INTI International University, Nilai, Negeri Sembilan, MY; 4 International Consultant, Petrosphere Inc, Puerto Princesa, 5300 Palawan, PH

## Abstract

**Background and Purpose:**

Musculoskeletal disorders (MSDs) are alarmingly high among migrant workers in Malaysia. MSDs are the most prevalent occupational-related conditions in most parts of the world affecting function, productivity and overall health-related quality of life. Therefore, this study aims to determine the profile of Filipino migrant workers in Malaysia and their various musculoskeletal complaints.

**Method:**

This study utilized a quantitative, nonexperimental, cross-sectional research design. A total of 60 subjects were randomly selected after passing the study’s sampling criteria. The Nordic Musculoskeletal Questionnaire (NMQ) was to used to determine common MSDs affecting the various regions in the body. The Demographic Pofile Sheet was provided to gather a subject’s demographic characteristics.

**Results:**

Filipino migrant workers mostly complain of pain in the low back area (60%) and shoulder pain (60%), followed by pain in the upper back (48.3%) and neck pain (45%) in the last 12 months. Household workers accounting for 73.3% of the subjects commonly complain of pain in the hips/thighs (78.9%), while workers in the service industry commonly complain of knee pain (39.1%).

**Conclusions:**

Results imply that Filipino migrant workers have a higher prevalence of shoulder and lower back pain in the last 12 months. Household workers are more susceptible to hip/thigh pain. Interventions focusing on ergonomics policy implementation, education on posture and lifting techniques and physical function is recommended. Further studies should consider the psychological and psychosocial aspects of migrant employment, which are known risk factors for MSDs.

## Introduction

In 2015, more than 2.3 million Filipinos [1] were working overseas either in land-based or sea-based deployment. The Middle East and Asia remain as the two leading destinations among Filipino migrant workers, and Malaysia is the eighth top destination among land-based overseas Filipino workers (OFW) [5]. Household service workers remain the top land-based deployment, followed by manufacturing laborers and nursing professionals [1]. In Malaysia, an alarmingly high level of musculoskeletal pain is reported among manufacturing sector migrant workers. Pain related to musculoskeletal disorders has an overall incidence of 64.4% [2]. An overall 35% incidence of work-related musculoskeletal disorders among migrant workers were reported among Vietnamese, Thai and Filipino women working in South Korea [3]. Women generally report a higher incidence of pain (87%) than men (76%) related to musculoskeletal disorders (MSDs) [4]. Similarly, more women reported a high incidence of MSDs compared to men among elderly care workers [5].

Generally, it is reported that there is a 6–9% loss of productivity aside from the hidden costs of musculoskeletal disorders [4]. It generally results in an average loss of 1.5 working hours or a total of 4–5% lost in productivity. This could translate to an estimated additional operational cost of $25,200 per year in a medium-sized company [4]. Globally, the economic burden of work-related illnesses and fatalities is an estimated 1.8% to 6% of gross domestic product (GDP) [6].

Musculoskeletal disorders are the most prevalent occupational-related condition in Europe and in South Korea [7]. It is reported among workers under agricultural, construction, manufacturing, health and service sectors, which were attributed to complex ergonomic factors [7]. MSDs were believed to be due to a wide variety of factors generally categorized as physical and psychosocial. The biomechanical model suggests the correlation between task performed and mechanical load required in the performance of work. On the other hand, work-related stresses are associated with high reports of pain [2].

Pain and reduced physical function do not only affect productivity, but also lead to a substantial impact on health-related quality of life (HRQOL) [8]. “Pain is defined by the individuals who are experiencing it [9].” *Pain* may signify a severe type of pain, *Hurt* may mean a moderate discomfort, and an *Ache* means a minimal pain [10]. However, regardless of how pain is described or is presented in an individual, whether acute or chronic, mild or severe, it remains to have profound effects on human life, affecting sense of efficacy and social competence [11].

Lower back pain (LBP) is the most common musculoskeletal complaint among Filipinos and Vietnamese, followed by the shoulder region among all migrant workers in Korea [3]. Elderly care home workers in Malaysia reported a prevalence of 33.8% of back pain [12]. Myanmar migrant workers in a food processing industry in Thailand had a point prevalence of pain in the low back area of 28.5%. Factors identified as increasing the risk of back pain include age, poor general health, history of back pain, twisting posture and slippery floor [13]. Similarly, Cambodian fruit workers in Thailand had a prevalence of LBP of 41.3%, which was more common among women (44.7%) than men (38.9%). Aside from LBP, they likewise reported common MSD symptoms affecting the upper back (28.2%), neck (23.9%) and shoulder (21.6%) [14]. In the United States, hotel service workers and cleaners reported MSDs commonly affecting the lower back (63%), followed by the upper back (59%) and neck (43%), which were attributed to physical workload and ergonomic issues [15]. Pain in the low back area was found to be correlated with heavy physical work, heavy or frequent manual operations, repeated trunk rotation and prolonged sitting [2].

A systematic review among female factory workers in Sri Lanka reported a total prevalence of neck and shoulder pain of 55%. This was probably due to contextual attributes related with workforce, ergonomics and access to occupational health services [16]. Occupations requiring stooped postures, exposure to vibrations and jarring motions are more prone to chronic pain in the low back [17]. Service industry workers, such as call center operators, are prone to develop neck, shoulder, upper back and lower back pain owing to poor ergonomics, repetitive strain and static or monotonous posture in line with their work. Women call center agents have higher MSDs compared with men. MSDs were believed to be due to individual factors, working techniques, coping strategies and factors outside work, because both males and females perform the same tasks at work [18].

Manufacturing migrant workers reported common MSDs affecting the back, shoulders, neck and legs. Manual handling of heavy objects done during manufacturing processes include lifting, lowering, pushing, pulling and carrying. Manual tasks at or above shoulder level, static neck flexion, increased workload and poorly designed work stations predispose individuals to neck and shoulder pain [2]. The shoulder region presents the most prevalent MSDs among elderly care workers in Malaysia [19]. Hip and thigh has an overall prevalence of 32.7%, while a rate of 40.4% affects the ankles and feet among live-in practical nurses [19]. Knee problem is common among Thai furniture workers and those older than 50 years [20].

A growing body of literature suggests that musculoskeletal pain is associated with anxiety and depression, which are correlated with severe pain behavior [21]. Pain experience is complex and is affected by multiple factors, and perceptions of pain and behaviour are influenced by socio-cultural contexts of those experiencing it [10]. Female immigrant workers in the United States reported general feelings of displacement, loneliness and stress related with work, which are possible factors for a higher sensitivity to pain perception [22]. In addition, a systemic review in 2004 stated that populations with weak psychological constitutions aside from weak physiological health are more prone to develop work-related MSDs [23]. Therefore, MSDs may be common among migrant workers due to a lack of emotional support and additional psychological pressure to adapt to the new living and working environment.

However, in spite of numerous studies about MSDs in various industry-based occupations and among migrant workers, there is still a limited study about the profile of Filipino migrant workers in Malaysia with symptoms of MSDs. Studies about Filipino migrant workers coming from various employment sectors and their current musculoskeletal symptoms were likewise lacking. Therefore, the aim of this study is to determine the profile of Filipino migrant workers with work-related MSDS in Malaysia. Furthermore, it will attempt to establish specific regions of the body affected by MSDs, which may serve as a basis for an appropriate intervention plan.

## Methodology

### Research design

A quantitative approach, nonexperimental, cross-sectional study design was adapted to describe the profile of Filipino migrant workers with work-related musculoskeletal disorders. Three researchers collected, analysed and tabulated the data obtained using a demographic profile sheet and Nordic Musculoskeletal Questionnaire (NMQ). Questionnaires were administered by the researchers among the subjects and, if necessary, provided further information and/or clarification about the questions.

All informants were provided an informed consent form (ICF) and detailed information sheet about the nature and purpose of the study. Personal identity and privacy were observed at all times. The study was reviewed and approved by a local university’s ethical review panel.

### Research locale, population and sampling

The research was done in a local nongovernmental organization (NGO) for Filipino migrant workers based in Kuala Lumpur, Malaysia. After obtaining the necessary permissions, the subjects were screened using the demographic profile sheet and NMQ for identification of prospective informants with MSDs. All informants signed the ICF prior to the study.

Subjects were randomly selected from individuals identified as having complaints of musculoskeletal pain in any region of the body, having worked for at least 1 year in Malaysia, and having been continuously employed in a manufacturing, service-oriented or household service sector. Subjects with complaints of pain caused by other conditions, such as kidney disease, gall bladder problems, premenstrual syndrome, chest pains and other nonmusculoskeletal causes of pain, were excluded from the study. Out of 70 available subjects, only 60 subjects passed the inclusion and exclusion criteria.

### Research instruments

Demographic characteristics, such as age, sex, marital status, number of years working (as a migrant worker) and nature of job, were collected using a demographic profile sheet. The NMQ was used to screen subjects with musculoskeletal disorders. It has a sensitivity of 66–92% and specificity of 71–88% in detecting pain. It has been extensively used in assessing various musculoskeletal problems, such as those among computer and call center workers, car drivers, coopers, nursing and forestry workers to name a few. NMQ is a sensitive, repeatable and useful screening and surveillance tool for musculoskeletal disorders used across a variety of occupations [24].

### Data collection and analysis

Researchers administered and collected all relevant data from a single visit to the NGO office. Information relevant to the questions was given to ensure that subjects understood the nature of the study. All data were analyzed using descriptive statistics, such as mean, mode and frequency distribution, while standard deviation was used as a measure of dispersion. Statistical Package for Social Sciences (SPSS) Version 10 was used for quantitative analysis.

## Results

There were 80 questionnaires distributed, out of which only 70 response sheets were returned, yielding an 87.5% response rate. A total of 70 subjects were initially screened for their eligibility to participate in the study. Ten subjects were excluded after failure to meet the sampling criteria. Data from 60 subjects were sent for final analysis. The demographic characteristics of the subjects are summarized in Table 1.

**Table 1:** Subjects’ Demographic Characteristics.

Variables	Values

Age (in years, Mean ± SD)	37.53 ± 9.41
Sex (# of Females)	54 (90%)
Years of Working (Mean ± SD)	8.05 ± 7.74
Marital Status	
Single	25 (41.7%)
Married	21 (35%)
Separated	9 (15%)
Widow/Widower	5 (8.3%)
Nature of Work	
Household Work	44 (73.3%)
Service Industry	13 (21.7%)
Manufacturing	3 (5%)

A majority of Filipino migrant workers are Female (90%), with a mean age of 37.53 ± 9.41 years and an average of 8.05 ± 7.74 years working in Malaysia. Most Filipino migrant workers are single (41.7%) and belong to the household work sector (73.3%).

The work-related MSDs among Filipino migrant workers are summarized in Table 2. Pain in the last 12 months signifies the history of musculoskeletal symptoms in the past year. The impact on work performance in the past 12 months owing to the presence of MSDs reflects the effect of MSDs on productivity. Presence of pain in the past 7 days signifies the presence of an acute MSD requiring immediate attention.

**Table 2:** Work-Related Musculoskeletal Disorders of Filipino Migrant Workers.

Body Regions	Pain in the last 12 months	Impact on Work Performance	Pain in the last 7 days

Neck	27 (45%)	18 (30%)	8 (13.3%)
Shoulder	36 (60%)	19 (31.7%)	14 (23.3%)
Right	10 (16.7%)		
Left	1 (1.7%)		
Both	25 (41.7%)		
Elbow	15 (25%)	8 (13.3%)	7 (11.7%)
Right	7 (11.7%)		
Left	1 (1.7%)		
Both	7 (11.7%)		
Wrist/Hand	26 (43.3%)	19 (31.7%)	10 (16.7%)
Right	11 (18.3%)		
Left	4 (6.7%)		
Both	11 (18.3%)		
Upper Back	29 (48.3%)	17 (28.3%)	14 (23.3%)
Lower Back	36 (60%)	18 (30%)	12 (20%)
Hips/Thighs	19 (31.7%)	12 (20%)	9 (15%)
Knees	23 (38.3%)	17 (28.3%)	10 (16.7%)
Ankles/Feet	17 (28.3%)	18 (30%)	7 (11.7%)

Filipino migrant workers mostly complained of pain in the low back area (60%) and shoulder pain (60%), followed by pain in the upper back (48.3%) and neck pain (45%) in the last 12 months. Subjects with pain in the shoulder (31.7%) and wrists/hand (31.7%) have reported a relatively high impact on work performance in the last 12 months, followed by pain in the neck (30%), lower back (30%) and ankles/feet (30%). Surprisingly, all of the subjects reported pain in the last 7 days, with most of them complaining of pain in the shoulder (23.3%) and upper back region (23.3%).

Work-related MSDS among Filipino migrant workers by nature of their job is shown in Table 3.

**Table 3:** Work-Related Musculoskeletal Disorders of Filipino Migrant Workers per Nature of Job in the Last 12 Months.

Body Regions	Household Work*	Service Industry*	Manufacturing Industry*	Total

Neck	18 (66.7%)	7 (25.9%)	2 (7.4%)	27 (45%)
Shoulder	26 (72.2%)	9 (25%)	1 (2.8%)	36 (60%)
Elbow	11 (73.3%)	3 (20%)	1 (6.7%)	15 (25%)
Wrist/Hand	17 (65.4%)	7 (26.9%)	2 (7.7%)	26 (43.3%)
Upper Back	21 (72.4%)	7 (24.1%)	1 (3.4%)	29 (48.3)
Lower Back	23 (63.9%)	11 (30.6%)	2 (5.6%)	36 (60%)
Hips/Thighs	15 (78.9%)	2 (10.5%)	2 (10.5%)	19 (31.7%)
Knees	13 (56.5%)	9 (39.1%)	1 (4.3%)	23 (38.3%)
Ankles/Feet	10 (58.8%)	5 (29.4%)	2 (11.8%)	17 (28.3%)

*HOUSEHOLD WORK includes domestic helpers, personal drivers, gardeners, caregivers, live-in practical nurses and related work; SERVICE INDUSTRY includes service crews, waiters/waitresses, cashiers, janitors/cleaners, receptionists, chambermaids/room service, customer care, maintenance work; MANUFACTURING industry work includes factory workers, food processing work, assembly work, construction workers and laborers, tinsmiths, foundry personnel, sewers.

Household workers reported a high prevalence of musculoskeletal pain at the hips/thighs (78.9%), followed by elbows (73.3%), upper back (72.4%) and shoulder (72.2%). Migrant workers belonging to the service industry reported a high prevalence of MSDs of the knees (39.1%), followed by lower back (30.6%). Whereas, those in the manufacturing industry reported pain affecting the ankles/feet (11.8%), followed by pain in the hips and thighs (10.5%).

## Discussion

A majority of the Filipino migrant workers deployed in Malaysia are female, young adults who were employed in household work similar to the data presented in 2014–2015 [1]. Compared with other sectores, women were generally employed for household work and are known to be at higher risk for MSDs [4]. Similarly, there are more Cambodian women who reported a higher prevalence of MSDs in the fruit processing sector [14].

All Filipino migrant workers worked on average more than eight years, which is quite different from the study conducted in 2012, which stated that those who were working for more than five years are less likely to develop MSDs due to their level of knowledge on workplace safety [5]. The high prevalence of MSDs may be explained by the repetitive and heavy tasks performed and the mechanical load adding more stress on the body both statistically and dynamically as proposed by the biomechanical model [2].

Pain related to MSDs affecting the shoulder is significantly higher among Filipino migrant workers in Korea (3.8%) [3], among Cambodian fruit workers (21.6%) [14] and manufacturing industry workers (55%) from Sri Lanka [16]. Heavy manual tasks, high workload and poorly designed work stations are known factors for shoulder pain [2]. Workers with pain in the shoulder and wrist/hand are often those involved with higher repetitive work in an awkward posture [25], which can have a significant impact on job performance, especially for those involved with manual tasks.

Low back pain is reported to be significantly higher compared to a study conducted among Filipinos and Vietnamese migrant workers in Korea (35%) [3], elderly care home workers in Malaysia (33.8%) [12], Myanmar manufacturing workers (28.5%) [13], and Cambodian fruit processing workers (41.3%) [14] in Thailand. However, the overall rate of pain in the lower back region is slightly lower than hotel services workers in the United States (63%) [14]. Heavy work demands, repeated trunk rotation and tasks requiring lifting, lowering, pushing, pulling and carrying heavy objects were found to be highly correlated with a high risk of back pain [2]. Poor body mechanics, such as a stooped posture, increases muscular and ligamentous tension at the back, increasing the risk of LBP [17]. Tasks involving such bodily movements are common among those involved with household work, manufacturing and service-oriented sectors.

Filipino migrant workers reported a relatively higher pain rate in the upper back compared with fruit workers (28.2%) from Cambodia [14]. Upper back problems are common in service industry workers due to poor ergonomics, repetitive strain and static or monotonous posture in line with their work [18].

Pain in the neck region among Filipino migrant workers is slightly higher among hotel service workers and cleaners in the United States (43%) [14] and among Cambodian fruit workers (23.9%) in Thailand [14]. However, it is lower than Sri Lankan manufacturing industry workers (55%) [16]. Manual tasks requiring the arms to be placed at or above the shoulder level, as well as static flexed posture of the neck, increases tension at the posterior neck structures, thereby predisposing an individual to neck pain [2]. Extended periods of work, insufficient recovery periods and rest, repetitive tasks and velocity of movement are found to be factors that increase the risk for MSDs of the upper limbs among workers involved in manual work [26].

Household workers reported a high prevalence of pain in the shoulder, elbows, upper back and hips/thighs. Similarly, migrant workers in the manufacturing sector commonly reported lower back pain. Upper limb pain is a common musculoskeletal complaint among industry workers and occupations requiring manual tasks. Ligaments and tendons are damaged by continuous or intermittent loading, leading to cumulative trauma and inflammatory responses, especially when stresses were applied continuously, thereby limiting the time needed for tissue repair [26], Hip/thigh pain are common among live-in practical nurses [20] owing to longer time spent standing and in tasks requiring lifting and other patient-related care.

Workers who belong to the service-oriented sector commonly reported knee and lower back problems. Similarly, back problems are common among hotel services workers in the United States (63%), which may be attributed to physical workload and ergonomic issues [15]. Knee problems are likewise common among care workers due to highly stressful job-related activities, with lack of rest and repetitive tasks involved with elderly care [5]. Workers who are required to stand for prolonged periods of time have a higher risk of developing back pain [27]. Filipino migrant workers in the manufacturing industry commonly reported pain in the ankle and foot region, which is similar among elderly care workers in Malaysia [5]. In addition, tasks requiring pushing, pulling and lifting are likewise related with a high incidence of ankle pain [5].

## Conclusions and Recommendations

In summary, the results of the study suggest that Filipino migrant workers have a higher prevalence of MSDs in the shoulder and lower back region. This study confirms that, whether in the last 12 months or in the last seven days, the shoulder and lower back region are more susceptible to work-related pain. Middle-aged women working in the household sector frequently complain of pain in the hip/thigh and the upper back region, which may be due to the physical and postural demands of their occupation. Occupations requiring long periods of standing, lifting and carrying are more prone to back pain; whereas, occupations requiring repetitive manual tasks are more susceptible to upper limb pain involving the shoulder, elbows, wrists and hands.

Moreover, the Guidelines on Ergonomics Risk Assessment at Workplace 2017 by the Department of Occupational Safety and Health, Ministry of Human Resource Malaysia [28] should be fully implemented, especially for foreign workers. Under this, ergonomics risk assessment (ERA) should be conducted to reduce cases of occupational diseases that indirectly affect labor productivity, profitability and cost of compensation. A policy on adherence to occupational safety and health regulations should be enforced and extended to service industry workers.

A systematic and comprehensive objective approach and treatment should address biomechanical and physiological issues, as well as the ergonomic design of the workplace among Filipino migrant workers in Malaysia. Educational interventions, such as rest intervals, proper lifting, carrying and body posture, should be given to address postural- and movement-related disorders of the body to reduce the risk of work-related MSDs. An intervention program to improve muscle strength, endurance and flexibility may be given in the form of a home exercises program that migrant workers can easily perform during their free time and offers no additional cost to the worker and employer.

Further studies should consider the psychological and psychosocial aspects of migrant employment, because these are known risk factors for MSDs. In addition, the impact of MSDs on migrant workers’ health-related quality of life warrants further investigation.
